# Traditional chest drainage *versus* drainage by thoracotomy: a prospective randomized study

**DOI:** 10.31744/einstein_journal/2020AO4409

**Published:** 2019-10-03

**Authors:** Thiago Gangi Bachichi, Luiz Eduardo Villaça Leão, João Alessio Juliano Perfeito, Andre Miotto, Caio Santos Holanda, Altair da Silva Costa

**Affiliations:** 1 Universidade Federal de São Paulo São PauloSP Brazil Universidade Federal de São Paulo, São Paulo, SP, Brazil.

**Keywords:** Drainage, Thoracotomy, Thoracic surgery, Pain

## Abstract

**Objective:**

To compare the chest tube drainage by the same thoracotomy intercostal space with the traditional approach in patients undergoing muscle-sparing thoracotomy.

**Methods:**

We evaluated 40 patients aged ≥18 years who underwent elective muscle sparing thoracotomies. Patients were divided into two groups of 20 patients. One group underwent thoracic drainage by the same intercostal space of thoracotomy and the other by traditional chest drainage approach.

**Results:**

The mean length of hospital stay for the intercostal drainage group in the intensive care unit was 1.5 day (1.0 to 2.0 days) and 2.0 days (25.1 to 3.0 days) for the traditional chest drainage group (p=0.060). The intercostal drainage group had mean length of hospital stay (p=0.527) and drainage (p=0.547) of 4 days, and the traditional chest drainage group and 2 and 5.5 days, respectively. Dipirona and tramadol doses did not differ between groups (p=0.201 and p=0.341). The mean pain scale values on first postoperative was 4.24 in the drainage by the same intercostal group and 3.95 in the traditional chest drainage (p=0.733). In third postoperative day, mean was 3.18 for the first group and 3.11 for the traditional group (p=0.937). In the 15^th^ day after surgery, drainage by the incision was 1.53 and the traditional chest drainage was 2.11 (p=0.440), 30^th^ days after drainage by incision was 0.71 and traditional chest drainage was 0.84 (p=0.787). Complications, for both groups were similar with 30% in proposed drainage and 25% in traditional approach (p=0.723).

**Conclusion:**

Drainage by the same thoracotomy intercostal space was feasible and results 30 days after surgery were not inferior to those of the traditional chest drainage approach.

## INTRODUCTION

Thoracotomy is still a widely used approach although the advances in videothoracoscopy. This approach advantages are related with excellent exposition of structures, which is the one used to access large tumors. On the other hand, major disadvantages are the long duration of the procedure, the high intensity of surgical pain, the need of large incisions, and the high incidence of seroma, and surgical wound infection.^1 , 2^

New techniques have been created to reduce complications, such as muscle-sparing thoracotomy that may avoid resection of serratus anterior muscle and the large dorsal muscle.^3 , 4^ Some other techniques associated with muscle-sparing procedure may reduce pain after the surgery. One of these techniques is preservation of neurovascular intercostal bundle that, in a prospective and randomized study including 40 patients, presented lower mean values in analogic visual pain scale than the group in which the bundle was not preserved.^5^

For decades, we believed in the dogma of the need of additional intercostomy to introduce chest drainage in the end of the surgery, and this belief was justified by supposed additional infection risk in surgical incision. In the last years, a number of techniques have appeared suggesting that chest drainage can be inserted in the same intercostal space incision. This led to the possibility of drainage using thoracotomy including open surgical cases.

## OBJECTIVE

To compare chest drainage by the same thoracotomy intercostal space to traditional chest drainage in patients undergoing muscle-sparing lateral thoracotomy.

## METHODS

**Figure 1:**
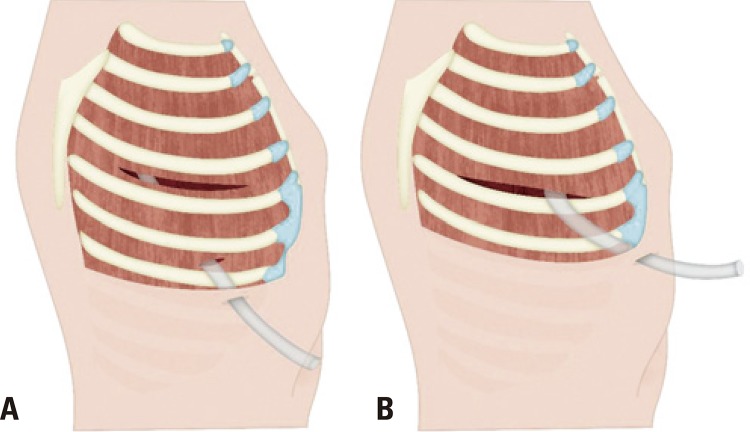
Chest drainage. (A) traditional chest drainage; (B) drainage using the same intercostal space thoracotomy (DI)

This prospective and randomized clinical trial was conducted at a single hospital center. The study was approved by the ethical and research committee, number 733,049, CAAE: 32193614.6.0000.5505. All patients were informed about the objective of the study and received the evaluation of results after the surgery. The consent term was approved by the Ethical Committee in Research. The study was blind and included patients who were candidate to lateral thoracotomy or who had previously conducted video-assisted thoracoscopic surgery, urgent and emergent surgeries, placement of more than one chest drain, resection of chest wall, had kidney failure or severe cardiac failure, and who underwent radiotherapy and chemotherapy. Sample size calculation was based on analogic visual pain scale in preservation of intercostal bundle group in a study previously conducted by our service: 4, 12, with standard deviation of 2.63.^5^ The number of controls per case was 1, and the expected response was a difference of 40%, which resulted in minimal sample size of 18 patients per group. For this reason, patients were separated in 10 blocks with 4 participants in each block, and they were included into two groups composed by 20 patients in randomized format and on-line web-based randomization page (www.randomization.com). In a group, the drainage was conducted using the same intercostal space thoracotomy (DI), figure 1A , and, the other group underwent the traditional chest drainage (DT).

In patients who underwent DI, an anterior accessory incision “counter-opening” type measuring 4m away from the main incision was carried out. Subsequently, a chest drain (28F) was also inserted under the right side view, in the same intercostal space used for thoracotomy. In the end of the surgery, patients who underwent DI performed a new incision measuring around 1cm on two intercostal spaces below the thoracotomy, and this incision was done in opening plan using a Kelly or Rochester forceps, followed by passage of pleural drain (28F) under direct view.

All individuals included in the study were evaluated after the surgery. Data collection included patients’ previous diseases and surgeries, medications, age, sex, ethnic, body mass index (BMI), smoking habit, smoking load and forced expiratory volume in the first second (FEV1) before surgery among patients who underwent the exam. Patients underwent pre-anesthesia induction before the epidural catheter insertion. Processes of the cavity opening and closing were standardized to in an attempt to isolate possible variables.

During surgery, we collected data such as surgical time (in minutes), type of procedure, presence of bleeding during the procedure and total volume in milliliters, possible fracture of the rib, and need of intraoperative blood transfusion. Patients were followed-up after surgery, data were collected on all days of hospitalization and in the first and second outpatient follow-up consultation that occurred 15 and 30 days after the surgery.

We recorded dosages of intravenous and oral analgesic during hospitalization as well as the amount of analgesic solution infused using an epidural catheter that consisted of 16mL of bupivacaine 0.25%, including 4mL of fentanyl 50mcg/mL. We also recorded information about presence of complications and the day they occurred. Post-operative complications were subcutaneous emphysema, hemorrhage, pneumonia, surgical wound infection, residual pleural cavity greater than 4cm for more than 1 week, cardiovascular complications, prolonged shortness of breath (over 5 days), dehiscence of surgical wound, pleural empyema, symptomatic pleural effusion, atelectasis, worsening of ventilation standard and any other complications related to surgery. Time of complication consisted of assessment of the day in which complication was noticed for the first time, and the presence of any complication in the operative period.

We also measured the number of days that patients remained in the intensive care unit and in the hospital, and also total debit drainage in mL. Pain measure was assessed by pain scale from 0 to 10 in the post-operative period in the first, third, fifteenth and thirtieth day after the surgery.

### Statistical analysis

Descriptive analyses for quantitative data that presented normal distribution were conducted using means followed by appropriate standard deviations. Quantitative data without normal distribution were expressed by means and interquartile intervals (IQI, 25-75%). Shapiro-Wilk test was used to evaluate normal distribution presupposes in each group and Levene’s test to obtain homogeneity of variances between groups. Categorical variables were expressed by their frequencies and percentages.

For the analyses of two factors (group and time), we used the analysis of variance of two-factor for repeated-measures for single factor (time). The Bonferroni test was used when comparisons of multiple medians were required. We applied the Student’s *t* test when two measures were compared. Mann-Whitney non-parametric test was used to assess variables that normal distribution was not observed. The chi-squared test and Fisher’s exact test were used to compare proportions of categorical variables between groups.

Type I error (α) of 0.05 probability was considered in all inferential analyses. Descriptive statistical analysis and inferential were performed using the (SPSS) software 21.0 for Windows.

## RESULTS

In the assessment of homogeneity of groups, we observed statistically significant difference only in mean value of BMI, which was higher in the DI Group (p=0.029). Group features were similar regarding age, pre-operative FEV1, sex, ethnic and comorbidities. Data collected during surgery showed that both groups were similar, each group included 20 patients, without significant differences concerning surgical time and hemorrhage during the procedure. Only one patient from each group (5%) had rib fracture during muscle-sparing approach using the Finochietto instrument (p<0.999). Individuals underwent three types of resection (bullectomy, non-anatomic resection and lobectomy) without observe statistical differences ( Table 1 ).

**Table 1:** Analgesia after surgery

	DI Group (n=20)	DT Group (n=20)	p value
Anesthetic dose through catheter* (mL)	59.8±43.33	54.0±23.37	0.60
Metamizole ^†^ (g)	5.0 (0-15.5)	10.0 (2.5-19.75)	0.201
Tramadol^†^ (mg)	100.0 (0-575.0)	300.0 (0-875.0)	0.341

Results expressed by means±standard deviations or median (interquartile interval P25-P75%). * Mann-Whitney test, ^†^ Student *t* test.

DI: drainage by using the same intercostal space thoracotomy; DT: traditional chest drainage.

**Figure 2:**
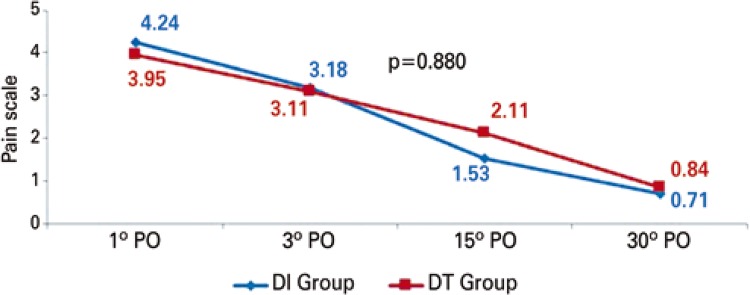
Graphic scale of post-operative pain (0 to 10). Comparison between means of the two groups throughout time

**Figure 3:**
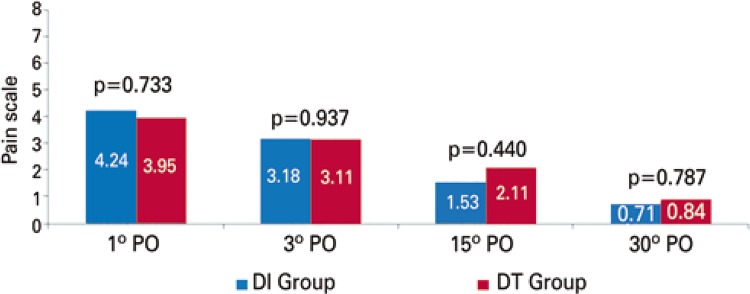
Graphic scale of post-operative pain, with mean and standard deviation of the two groups in each period

**Figure 4:**
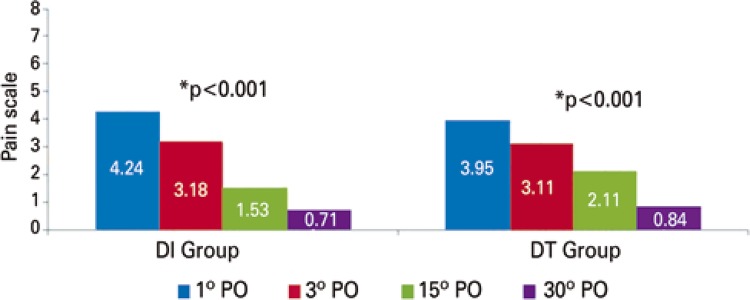
Graphic of scale of post-operative pain (means from 0 to 10). Individual analysis of each group through the time in predetermined days

In the pain assessment in predetermined days there were no statistically relevant distinction in the two groups (p=0.880). Figures 2 and 3 showed this progress in pain scale in each group and their comparison throughout time. In two approaches an important differences occurred concerning the improvement of pain throughout time. Figure 4 shows that two groups were analyzed independently, we also observed a drop in mean pain scale values for each group in the days that they were evaluated.

Analysis of variance (ANOVA). PO: post-operative; DI: drainage by using the same intercostal space thoracotomy; DT: traditional chest drainage.

Analysis of variance (ANOVA). PO: post-operative; DI: drainage by using the same intercostal space thoracotomy; DT: tradicional chest drainage.

Bonferroni correction. PO: post-operative; DI: drainage by using the same intercostal space thoracotomy; DT: traditional chest drainage.

Most frequent complications were atrial fibrillation and prolonged shortness of breath ( Table 2 ). In one patient from DT Group residual pleural cavity persisted up to 15^th^ day after surgery (p=0.311). Two patients from DI group died during the 30^th^ days follow-up, without statistical significance (p=0.487). One patient who were hospitalized for biopsy of interstitial lung disease did not present intercurrences until the fifth day after the surgery, however an worsening of ventilator standard and suggestive radiography of lung fibrosis were observed. The patient ended-up dying in seventh day after the surgery, although this treated with corticoids in pulsotherapy. Another patient, aged 65 years, who had chronic obstructive pulmonary disease and low functional reserve (FEV1: 45%) and was hospitalized because of nodular injury resection (adenocarcinoma), was readmitted to emergency room 16 days after the surgery reporting fever for 3 days. The diagnosis was pleural empyema, and the fluid was promptly drained.

**Table 2:** Complications per group

Complications	DI group N (%)	DT group N (%)	p value
Atrial fibrillation^†^	2 (10)	0	0.487
Prolonged shortness of breath^†^	0	2 (10)	0.487
Wall infection*	1 (5)	1 (5)	>0,999
Subcutaneous emphysema*	1 (5)	1 (5)	>0.999
Empyema^†^	1 (5)	0	0.311
Atelectasis^†^	0	1 (5)	0.311
Standard worsening of interstitial disease^†^	0	1 (5)	0.311
Deaths	2 (10)	0	0.487

* χ^2^ test; ^†^ Fisher test.

The patient evolved with worsening in clinical picture during hospitalization, skeptical shock and death on the 30^th^ day after the surgery.

## DISCUSSION

This study evaluated a new form of chest drainage. The drain was inserted in the same intercostal space of sparing thoracotomy incision in patients who underwent elective surgery. As a result, we did not find statistical differences in post-operative data such as duration of hospitalization, drainage, pain scale, and complications. In addition, non-inferiority margin was observed in proposed technique compared with DT.

The two groups were evaluated concerning a number of pre-operatory variables to seek any statistical significant that could compromise the interpretation of results. The only variable that presented statistical difference was the BMI. However, this fact may not have a practical effect and does not influence results, especially considering that this difference between means of BMI was low (only 2.45kg/m^2^). In other characteristics evaluated (age, sex, comorbidities and pre-operatory FEV1) there were no significant differences, which demonstrated that both groups, DI and DT, were homogenous. No differences between two groups related to intraoperative data were observed, given that surgical time, presence and means of hemorrhage volume were similar. This was expected because the two techniques are only related to drainage, which is always performed in the end of the surgery, right before the opening, which not interfere bleeding risks or intraoperative complications. In addition, the fact that proposed technique does not extend the surgical time is important for not increasing risks already linked to long surgeries.

Although many surgeons do not use the same thoracotomy incision for drainage due to fear of increasing infection rates of surgical wound, such problem was not observed in our study. Other factor that could restrict this option of drainage would be the possible increasing in risk of drains blockage. However, we did not observe an increasing in complications in DI Group, and this drainage was as effective as DT. No increasing was seen in infection rate of surgical wound, and there we no need of change drainage or request additional one.

Patients of our study who underwent DI, had mean hospitalization rate of only 4 days, without statistical difference with DT Group, which represented low mean time of hospitalizations among patient who underwent lung resections. Athanassiadi et al., study related to drainage volume reported total debt of 536±257mL, *i.e* ., lower than mean of DI Group (875mL).^6^ Some differences in studies may influence drainage volume. In our study, we used only one drain, and in the Athanassiadi et al. study, they used two drains.

Mean time of DI Group with drain was 4 days, while the mean of other studies was 4.7 days.

Pain reduction throughout the post-operative period was already observed in the literature.^5^

A retrospective analysis between uniportal VATS and three incisions were lower than pain scale score in the immediate post-operative period (4.95±0.38 *versus* 6.44±0.39; p=0.012) and in the first post-operatory (2.74±0.34 *versus* 3.78±0.35; p=0.039) and in the third post-operatory (1.32±0,20 *versus* 1.94±0.21; p=0.037). In DI Group, the mean pain scale of the first post-operative was similar (mean of 4.24), but this mean presented a pain reduction in the third post-operative (mean of 3.18) than patients who underwent minimally invasive techniques.^7^ Results of published studies allow the assumption that reduction in number of incisions can provide a pain reduction in the first days after the procedure. A retrospective Chinese study of 2015, the group was submitted to uniportal VATS that presented lower pain in analogic scale(3.6±0.7) than the group who underwent surgery through three incisions (5.5±1.0), p<0.05.^8^

Athanassiadi et al.^6^ study showed that patients who underwent muscle-sparing lateral thoracotomy had a mean of 1.4±0.9 in pain scale in the 30th day after the surgery. In our study, patients of two groups reported lower means (0.84 in DT and 0.71 in DI). Great means in pain scales of patients who underwent open surgeries were due to need of costal sparing with Finochietto instrument because of the compression of intercostal nerve. Drainage by the same incision can take advantage of the same intercostal space used and avoids compression by the drainage of other neurovascular bundle. Chronic pain was not evaluated in our study, for this reason, it would be necessary more time of follow for patients.^9^

In both studied groups, DI and DT, complications occurred mainly in the first week after the surgery, and these results were similar to data in searched literature.^10 , 11^

In our study, global complication rate was 27.5% and respiratory complication rate was 5%. In published literature, the prolonged shortness of breath occurred, on average, in only 1.9% of patients, and the lower observance in the study was 5%. We presented chest wall infection rate of 5% in each group, which was similar to those reported studies of the literature such as the reported by Akçali et al.,^4^ that found a rate of 3.3%, also among patients who underwent muscle-sparing lateral thoracotomy. Our two groups presented equal rates showing that proposed technique did not increase the risk of wound infection.

Two deaths occurred, but no statistical significance was found. These two patients had severe comorbidities, and their clinical picture worsened in the post-operative period. We believe that death causes were not related to complications of drainage.

The lack of statistically significant complications in groups confirmed the expectation, given that uniportal technique already used this type of drainage without problems compared with multiportal VATS.^12 , 13^ Our results show the safety of this technique and also that drain is still accessible when inserted in the proposed model. Drainage by using the same intercostal space allows preservation of other space that would be opened. In addition, this approach avoids the risk of injury to a second neurovascular bundle and also provides another incision in muscle layer.

In our study, the follow-up period of patients was short, only 30 days. For this reason, a longer follow-up is required to confirm if results of DI would be similar to those of DT regarding post-operative morbidity. A longer follow-up would also contribute to enable a comparison of chronic pain rate in both groups. The number of patients included in our study (only 40), did not allow to establish any regarding the superiority of the technique in comparison with DT.

## CONCLUSION

Results of chest drainage by thoracotomy was not inferior to results achieved by traditional drainage in patient who underwent muscle-sparing lateral thoracotomy. We did not observe increase in length of hospital stay, drainage time, drainage debt, pain scale, use of analgesics and complications in comparison with traditional drainage.

## References

[1] 1. López SS, Cacho MV, Muñoz PM, Merchán RJ. Incisiones y vías de abordaje quirúrgicas. Arch Bronconeumol. 2011;47(8):21-5. 10.1016/S0300-2896(11)70063-1 23351517

[2] 2. Dürrleman N, Massard G. Posterolateral thoracotomy. Multimed Man Cardiothorac Surg. 2006;2006(810):mmcts.2005.001453. 10.1510/mmcts.2005.001453 24412938

[3] 3. Ziyad S, Baskent A, Tanju S, Toker A, Dilege S. Isokinectic muscle strength after thoracotomy standard vs. Muscle-sparing posterolateral thoracotomy. Thorac Cardiovasc Surg. 2010;58(5):295-8. 10.1055/s-0030-1249829 20680907

[4] 4. Akçali Y, Demir H, Tezcan B. The effect of standard postolateral versus muscle-sparing thoracotomy on multiple parameters. Ann Thorac Surg. 2003;76(4):1050-4. 10.1016/s0003-4975(03)00565-4 14529983

[5] 5. Marchetti-Filho MA, Leão LEV, Costa-Junior AS. O papel da preservação do nervo intercostal no controle da dor aguda pós-toracotomia. J Bras Pneumol. 2014;6(4):470-9.

[6] 6. Athanassiadi K, Kakaris S, Theakos N, Skottis I. Muscle-sparing versus posterolateral thoracotomy: a prospective study. Eur J Cardiothorac Surg. 2007;31(3):496-9; discussion 499-500. 10.1016/j.ejcts.2006.12.012 17236781

[7] 7. Tamura M, Shimizu Y, Hashizume Y. Pain following thoracoscopic surgery: retrospective analysis between single-incision and three-port video-assisted thoracoscopic surgery. J Cardiothorac Surg. 2013;8:153. 10.1186/1749-8090-8-153 PMC3691684 23759173

[8] 8. Zhu Y, Liang M, Wu W, Zheng J, Zheng W, Guo Z, et al. Preliminary results of single-port versus triple-port complete thoracoscopic lobectomy for non-small cell lung cancer. Ann Transl Med. 2015;3(7):92. 10.3978/j.issn.2305-5839.2015.03.47 PMC4430732 26015934

[9] 9. Hopkins KG, Hoffman LA, Dabbs AV, Ferson PF, King L, Dudjak LA, et al. Postthoracotomy Pain syndrome following surgery for lung cancer: symptoms and impact on quality of life. J Adv Pract Oncol. 2015;6(2):121-3. Review. 10.6004/jadpro.2015.6.2.4 PMC4601892 26649245

[10] 10. Lima VP, Bonfim D, Risso TT, Paisani DM, Fiore JF, Chiavegato LD, et al. Influence of pleural drainage on postoperative pain, vital capacity and six-minute walk test after pulmonar resection. J Bras Pneumol. 2008;34(12):1003-7. 10.1590/s1806-37132008001200004 19180334

[11] 11. Sengupta S. Post-operative pulmonary complications after thoracotomy. Indian J Anaesth. 2015;59(9):618-26. 10.4103/0019-5049.165852 PMC4613409 26556921

[12] 12. Scott WJ, Allen MS, Darling G, Meyers B, Decker PA, Putnam JB, et al. Video-assisted thoracic surgery versus open lobectomy for lung cancer: a secondary analysis of data from the American College of Surgeons Oncology Group Z0030 randomized trial. J Thorac Cardiovasc Surg. 2010;139(4):976-81. 10.1016/j.jtcvs.2009.11.059 20172539

[13] 13. Gonzalez-Rivas D, Fieira E, Delgado M, Mendez L, Fernandez R, Torre M. Uniportal video-assisted thoracoscopic lobectomy. J Thorac Dis. 2013;5(Suppl 3):S234-45. 10.3978/j.issn.2072-1439.2013.07.30 PMC3771611 24040531

