# Supplementary material for: Traditional chest drainage versus drainage by thoracotomy: a prospective randomized study
Source: Einstein (Sao Paulo). 2019 Oct 3;18:eAO4409. doi: 10.31744/einstein_journal/2020AO4409 (PMC6896604; doi:10.31744/einstein_journal/2020AO4409)
Supplement: Translation

# Translation: Drenagem torácica tradicional *versus* drenagem pela toracotomia: estudo prospectivo randomizado

**Authors:** Thiago Gangi Bachichi, Luiz Eduardo Villaça Leão, João Alessio Juliano Perfeito, Andre Miotto, Caio Santos Holanda, Altair da Silva Costa

**Affiliations:** 1 Brasil Universidade Federal de São Paulo, São Paulo, SP, Brasil.

**Keywords:** Drenagem, Toracotomia, Cirurgia torácica, Dor

## Abstract

**Objetivo:**

Comparar a drenagem torácica pela mesma intercostotomia à drenagem tradicional em pacientes submetidos à toracotomia poupadora lateral.

**Métodos:**

Foram avaliados 40 pacientes maiores de 18 anos submetidos a toracotomias poupadoras laterais eletivas. Eles foram separados em dois grupos de 20 pacientes cada, sendo um submetido à drenagem torácica pelo mesmo espaço intercostal da toracotomia e o outro à drenagem tradicional.

**Resultados:**

No grupo da drenagem pela mesma intercostotomia, a mediana de tempo de internação em unidade de terapia intensiva foi de 1,5 dia (1,0 a 2,0 dias) e de 2,0 dias (1,25 a 3,0 dias) na drenagem tradicional (p=0,060). As medianas do tempo de internação (p=0,527) e de drenagem (p=0,547) foram ambas de 4 dias, no primeiro grupo, e de 2 e 5,5 dias, no grupo com drenagem tradicional. As doses utilizadas de dipirona e de tramadol não apresentaram diferenças estatísticas entre os grupos (p=0,201 e p=0,341). As médias da escala de dor foram 4,24 no primeiro dia pós-operatório do grupo com a drenagem proposta e 3,95 nos drenados da forma tradicional (p=0,733); no terceiro pós-operatório, foi de 3,18 para o grupo drenado pela incisão e de 3,11 nos drenados da forma tradicional (p=0,937). No 15º dia após a cirurgia, a drenagem pela incisão foi de 1,53 e a tradicional de 2,11 (p=0,440); no 30º pós-operatório, foi de 0,71 e 0,84, respectivamente, para a incisão e a forma tradicional (p=0,787). Em relação às complicações, os grupos foram semelhantes, com 30% na drenagem proposta e 25% na drenagem tradicional (p=0,723).

**Conclusão:**

A drenagem pelo mesmo espaço intercostal foi exequível e não apresentou inferioridade à técnica tradicional no período pós-operatório estudado de 30 dias.

## INTRODUÇÃO

A toracotomia ainda é uma via muito utilizada, apesar dos avanços relacionados à videotoracoscopia. Suas vantagens decorrem da excelente exposição das estruturas, sendo o acesso de escolha para grandes tumores, por exemplo. Por outro lado, suas maiores desvantagens são o tempo prolongado de procedimento, a maior intensidade de dor operatória, a necessidade de grandes incisões, e a maior incidência de seroma e de infecção de ferida operatória.^1 , 2^

No intuito de reduzir essas complicações, foram criadas novas técnicas, como a toracotomia poupadora de músculo, evitando a secção do músculo serrátil anterior e do grande dorsal.^3 , 4^ Algumas outras técnicas associadas à cirurgia poupadora podem diminuir a dor no período pós-operatório. Uma delas é a preservação do feixe intercostal neurovascular, que, em estudo prospectivo e randomizado com 40 pacientes, apresentou valores médios menores na Escala Visual Analógica de Dor do que o grupo em que o feixe não foi protegido.^5^

Por décadas, acreditávamos no dogma da necessidade de uma intercostotomia adicional para introdução do dreno torácico ao final da operação, sendo esta justificada pelo suposto risco adicional de infecção na incisão operatória. Nos últimos anos, surgiram várias novas técnicas, nas quais a drenagem torácica se dá pelo mesmo espaço intercostal utilizado no acesso. Isso suscitou a possibilidade da realização da drenagem pela toracotomia também na cirurgia aberta.

## OBJETIVO

Comparar a drenagem torácica pela mesma intercostotomia à drenagem tradicional em pacientes submetidos à toracotomia poupadora lateral.

## MÉTODOS

Este ensaio clínico prospectivo, randomizado, foi realizado em um único centro hospitalar e aprovado pelo Comitê de Ética em Pesquisa conforme parecer número 733.049, CAAE: 32193614.6.0000.5505. Todos os pacientes foram orientados sobre os objetivos do estudo e quanto à avaliação dos resultados pós-operatórios. O Termo de Consentimento foi aprovado pelo Comitê de Ética em Pesquisa. O estudo foi cego em pacientes candidatos à toracotomia poupadora lateral eletiva, uma vez que eles não tinham conhecimento até o final da avaliação se o tipo de drenagem realizado era o tradicional ou o proposto.

Foram incluídos pacientes com idade ≥18 anos candidatos à toracotomia poupadora lateral eletiva. Já os critérios de exclusão foram o uso prévio de opioides, diagnóstico de síndrome de dor crônica, toracotomias ou VATS ( *Video-assisted thoracoscopic surgery)* prévias, operações de urgência e emergência, colocação de mais de um dreno torácico, ressecção de parede torácica, insuficiência renal ou cardíaca severas, quimioterapia ou radioterapia prévias. O cálculo do tamanho da amostra foi realizado com base no maior valor da Escala Visual Analógica de Dor pós-operatória no grupo de preservação do feixe intercostal em trabalho realizado previamente em nosso serviço: 4,12, com desvio padrão de 2,63.^5^ O número de controles por caso foi 1, e a resposta esperada foi uma diferença de 40%, resultando em tamanho amostral mínimo necessário de 18 pacientes por grupo. Assim, os pacientes foram separados em 10 blocos de quatro participantes cada, tendo sido alocados nos dois grupos de 20 pacientes, de forma randomizada e *on-line* pela página *web-based randomization* (www.randomization.com). Em um grupo, foi realizada a drenagem pelo mesmo espaço intercostal da toracotomia (DI), representado na figura 1A , e, no outro, a drenagem tradicional (DT), representado na figura 1B .

**Figura 1:** Drenagem torácica. (A) Drenagem torácica tradicional; (B) Drenagem pelo mesmo espaço intercostal da toracotomia (DI)

Nos pacientes submetidos à DI, foi realizada incisão acessória anterior do tipo “contra-abertura” a 4cm da incisão principal. Assim, o dreno torácico 28F também foi introduzido sob visão direta, pelo mesmo espaço intercostal da toracotomia. Nos pacientes submetidos à DT, ao final da cirurgia, era realizada nova incisão de cerca de 1cm dois espaços intercostais abaixo da toracotomia, sendo realizada a abertura dos planos com uma pinça Kelly ou Rochester, seguida da passagem do dreno pleural 28F sob visão direta.

Todos os indivíduos incluídos passaram por avaliações pré-operatórias com coleta de dados, como doenças e cirurgias prévias, uso de medicamentos, idade, sexo, etnia, índice de massa corporal (IMC), presença de tabagismo, carga tabágica e volume expiratório forçado no primeiro segundo (VEF1) pré-operatório nos pacientes que realizaram o exame. Os pacientes foram submetidos à indução pré-anestésica pela equipe de anestesia e à inserção do cateter peridural. Os processos de abertura e fechamento da cavidade foram padronizados para isolar possíveis variáveis.

Durante a cirurgia, foram coletados dados como tempo cirúrgico em minutos, tipo de procedimento realizado, presença de sangramento durante o procedimento e volume em mililitros, eventual fratura de costela e necessidade de transfusão intraoperatória. Os pacientes foram acompanhados no período pós-operatório, com coleta de dados durante todos os dias de internação e no primeiro e segundo retornos ambulatoriais, que aconteceram 15 e 30 dias após a cirurgia respectivamente.

Durante a internação, foram anotadas as dosagens de analgésicos endovenosos e orais utilizados, bem como a quantidade da solução analgésica realizada pelo cateter peridural, que consistia em 16mL de bupivacaína 0,25%, acrescidos de 4mL de fentanil 50mcg/mL. A informação sobre a presença de complicações e o dia em que elas ocorriam também foi registrada. Consideramos complicações pós-operatórias: enfisema subcutâneo, hemorragia, pneumonia, infecção de ferida operatória, cavidade pleural residual maior que 4cm por mais de 1 semana, complicações cardiovasculares, perda aérea prolongada (maior que 5 dias), deiscência de ferida operatória, empiema pleural, derrame pleural sintomático, atelectasia, piora do padrão ventilatório e quaisquer outras complicações relacionadas à cirurgia. O tempo de complicação consistia na avaliação do dia em que era notada, pela primeira vez, a presença de alguma complicação no período operatório.

Também foram quantificados os dias de internação em unidade de terapia intensiva e no hospital, e o débito total drenado em mL. A aferição da dor no período pós-operatório foi realizada no primeiro, terceiro, décimo quinto e trigésimo dias pós-operatórios, por meio de uma escala de dor de zero a 10.

### Análise estatística

As análises descritivas para os dados quantitativos que apresentaram distribuição normal foram realizadas apresentando as médias acompanhadas dos respectivos desvios padrão. Os dados quantitativos sem distribuição normal foram expressos por medianas e intervalo interquartil (IQ; 25-75%). Os pressupostos da distribuição normal em cada grupo e a homogeneidade das variâncias entre os grupos foram avaliados, respectivamente, com o teste de Shapiro-Wilk e o teste de Levene *.* As variáveis categóricas foram expressas por suas frequências e percentagens.

Para análise de dois fatores (grupo e tempo), foi utilizada a análise de variância de duplo fator de medidas repetidas para um único fator (tempo). Quando foi necessário realizar comparações múltiplas de médias, foi usado o teste de Bonferroni. Quando foram comparadas duas médias, foi aplicado o teste *t* de Student para amostras independentes. Para variáveis em que não se verificou distribuição normal, recorreu-se ao teste não paramétrico de Mann-Whitney. Para comparação das proporções das variáveis categóricas entre os grupos, foram utilizados o teste do χ^2^e o teste exato de Fisher. Foi considerada probabilidade de erro do tipo I (α) de 0,05 em todas as análises inferenciais. As análises estatísticas descritivas e inferenciais foram executadas com o software (SPSS), versão 21, for Windows.

## RESULTADOS

Na avaliação da homogeneidade dos grupos, observamos diferença estatisticamente significativa somente no valor médio de IMC, que foi maior no Grupo DI (p=0,029). Em relação a idade, VEF1 pré-operatório, sexo, etnia e comorbidades, os grupos foram semelhantes. Nos dados colhidos durante a cirurgia, os dois grupos também foram semelhantes, cada um com 20 pacientes, sem diferenças significativas quanto a tempo cirúrgico e hemorragia durante o procedimento. Apenas um paciente de cada grupo (5%) apresentou fratura de costela durante o afastamento com o Finochietto (p<0,999). Os indivíduos foram submetidos a três tipos de ressecção (bulectomia, ressecção não anatômica e lobectomia) sem que houvesse diferenças estatísticas.

Também no período pós-operatório, não houve diferenças significativas em relação ao tempo de drenagem (p=0,547), tempo de internação em unidade de terapia intensiva (p=0,060), débito total pelo dreno (p=0,738) e tempo de internação em (p=0,527). A quantidade de analgésicos utilizada, incluindo o tramadol, a dipirona e a solução analgésica administrada pelo cateter peridural, foi aferida, e os grupos também não apresentaram diferenças estatísticas ( Tabela 1 ).

**Tabela 1:** Analgesia pós-operatória

	Gropo DI (n=20)	Grupo DT (n=20)	Valor de p
Dose de anestésico pelo cateter* (mL)	59,8±43,33	54,0±23,37	0,60
Dipirona^†^(g)	5,0 (0-15,5)	10,0 (2,5-19,75)	0,201
Tramadol^†^(mg)	100,0 (0-575,0)	300,0 (0-875,0)	0,341

Resultados expressos por média±desvio padrão ou mediana (intervalo interquartil P25-P75%). * Mann-Whitney;^†^teste *t* de Student.

DI: drenagem pelo mesmo espaço intercostal da toracotomia; DT: drenagem torácica tradicional.

Na avaliação da dor nos dias predeterminados também não houve distinção com relevância estatística quando comparados os dois grupos (p=0,880). As figuras 2 e 3 demonstram essa evolução da escala de dor em cada grupo e comparativamente, ao longo do tempo. Nas duas abordagens, ocorreram diferenças expressivas quanto à melhora da dor ao longo do tempo. Na figura 4 os dois grupos são analisados de forma independente. Nele, é possível observar a queda nos valores das médias da escala de dor de cada grupo ao longo dos dias avaliados.

**Figura 2:** Gráfico de escala de dor pós-operatória (zero a 10). Comparação entre as médias dos dois grupos ao longo do tempo

Análise de variância (ANOVA). PO: pós-operatório; DI: drenagem pelo mesmo espaço intercostal da toracotomia; DT: drenagem torácica tradicional.

**Figura 3:** Gráfico de escala de dor pós-operatória, com a média e desvio padrão, dos dois grupos, em cada período

Análise de variância (ANOVA). PO: pós-operatório; DI: drenagem pelo mesmo espaço intercostal da toracotomia; DT: drenagem torácica tradicional.

**Figura 4:** Gráfico de escala de dor pós-operatória (médias de zero a 10). Análise individual de cada grupo ao longo do tempo em dias predeterminados

* Teste de Bonferroni. PO: pós-operatório; DI: drenagem pelo mesmo espaço intercostal da toracotomia; DT: drenagem torácica tradicional.

As complicações mais frequentes foram fibrilação atrial e perda aérea prolongada, evidenciadas pela tabela 2 . Um paciente do Grupo DT persistiu com cavidade pleural residual até o 15º dia pós-operatório (p=0,311). Neste estudo, ocorreram dois óbitos durante o acompanhamento por 30 dias, ambos do Grupo DI, sem significância estatística (p=0,487). Um dos pacientes que internou para biópsia de doença intersticial pulmonar não apresentou intercorrências até o quinto dia pós-operatório, quando evoluiu com piora do padrão ventilatório e radiográfico sugestivo de fibrose pulmonar. Apesar do tratamento com corticoide em pulsoterapia, o paciente faleceu no sétimo dia pós-operatório. Outro paciente, com 65 anos, doença pulmonar obstrutiva crônica *Gold D* , com reserva funcional baixa (VEF1: 45%), internou para ressecção de lesão nodular (adenocarcinoma) e retornou no 16º dia pós-operatório ao pronto-socorro com história de febre em casa há 3 dias, sendo diagnosticado empiema pleural, prontamente drenado. Evoluiu com piora clínica durante a internação, choque séptico e faleceu no 30º dia pós-operatório.

**Tabela 2:** Complicações por grupo

Complicações	Grupo DI N (%)	Grupo DT N (%)	Valor de p
Fibrilação atrial^†^	2 (10)	0	0,487
Perda aérea prolongada^†^	0	2 (10)	0,487
Infecção de parede*	1 (5)	1 (5)	>0,999
Enfisema subcutâneo*	1 (5)	1 (5)	>0,999
Empiema^†^	1 (5)	0	0,311
Atelectasia^†^	0	1 (5)	0,311
Piora de padrão da doença intersticial^†^	0	1 (5)	0,311
Óbitos	2 (10)	0	0,487

* teste do χ^2^;^†^teste de Fisher.

## DISCUSSÃO

Este estudo avaliou uma nova forma de drenagem torácica, com o dreno sendo introduzido pelo mesmo espaço intercostal da toracotomia poupadora em pacientes submetidos às cirurgias eletivas. Como resultados, não foram encontradas diferenças estatísticas nos dados pós-operatórios, como tempo de internação e de drenagem, escala de dor e complicações. Com isso, não encontramos nenhum indício de inferioridade na técnica proposta em relação à DT.

Os dois grupos foram avaliados quanto às diversas variáveis pré-operatórias à procura de alguma significância estatística que pudesse comprometer a interpretação dos resultados. A única variável a apresentar diferença estatística foi o IMC. Contudo, esse fato não deve ter efeito prático nem influenciar nos resultados, uma vez que a diferença entre as médias de IMC era pequena (apenas 2,45kg/m^2^). Nas demais características avaliadas (idade, sexo, comorbidades e VEF1 pré-operatório), não houve diferenças significativas, o que demonstra que os dois Grupos, DI e DT, eram homogêneos. Também não houve qualquer diferença entre os dois grupos no que tange aos dados intraoperatórios, visto que o tempo de cirurgia, a presença e as médias de volume de hemorragia foram semelhantes. Isso era esperado, pois as duas técnicas dizem respeito apenas à drenagem, que sempre é realizada ao fim da cirurgia, logo antes do fechamento, não interferindo nos riscos de sangramento ou complicações intraoperatórias. Além disso, o fato de a técnica proposta não estender o tempo cirúrgico é importante, para não aumentar riscos já inerentes às cirurgias longas.

Apesar de muitos cirurgiões não utilizarem a mesma toracotomia para drenagem por receio do aumento nas taxas de infecção de ferida operatória, isso não foi observado neste estudo. Outro fator que poderia restringir essa opção de drenagem seria o possível aumento no risco de obstrução dos drenos. No entanto, não ocorreu aumento de complicações no Grupo DI, e esta drenagem se mostrou tão efetiva quanto a DT. Não observamos aumento na taxa de infecção da ferida operatória e nem necessidade de troca ou de drenagem adicional.

Nos pacientes do nosso estudo submetidos à DI, a média de internação foi de apenas 4 dias, sem diferença estatística com o Grupo DT, o que representa baixo tempo médio para internações em pacientes submetidos às ressecções pulmonares. Em relação ao volume drenado, Athanassiadi et al., apresentaram débito total de 536±257mL, ou seja, menor que a mediana do Grupo DI (875mL).^6^ Algumas diferenças entre os estudos podem ter influenciado no volume drenado. No nosso estudo, foi usado apenas um dreno e, no estudo de Athanassiadi et al., foram utilizados dois drenos. A mediana do Grupo DI foi de 4 dias com o dreno, enquanto a média desses autores foi de 4,7 dias.

A diminuição da dor ao longo do período pós-operatório já era observada na literatura.^5^ Uma análise retrospectiva entre a VATS uniportal e a realizada por três incisões mostrou menor pontuação na escala de dor no pós-operatório imediato (4,95±0,38 *versus* 6,44±0,39; p=0,012) e no primeiro (2,74±0,34 *versus* 3,78±0,35; p=0,039) e no terceiro pós-operatório (1,32±0,20 *versus* 1,94±0,21; p=0,037). No Grupo DI, a média na escala de dor do primeiro pós-operatório foi semelhante (média de 4,24), mas apresentou menor diminuição da dor no terceiro pós-operatório (média de 3,18) que estes pacientes submetidos a técnicas minimamente invasivas.^7^ Nesses estudos, é possível inferir que a diminuição no número de incisões pode proporcionar diminuição da dor nos primeiros dias após o procedimento. Em estudo retrospectivo chinês de 2015, o grupo que foi submetido a VATS uniportal apresentou menor dor na escala analógica (3,6±0,7) que o grupo submetido à cirurgia por três incisões (5,5±1,0), p<0,05.^8^

Em relação aos pacientes submetidos à toracotomia poupadora lateral, Athanassiadi et al.,^6^ apresentaram média de 1,4±0,9 na escala de dor no 30º pós-operatório. Já no nosso estudo, os pacientes dos dois grupos relataram médias menores (0,84 no DT e 0,71 no DI). As médias maiores nas escalas de dor dos pacientes submetidos a cirurgias abertas decorrem da necessidade do afastamento costal com o Finochietto, devido à compressão do nervo intercostal. A drenagem pela mesma incisão aproveita o mesmo espaço intercostal utilizado e evita a compressão pelo dreno de outro feixe neurovascular. A dor crônica não foi avaliada em nosso estudo; para isso, seria necessário mais tempo de acompanhamento dos pacientes.^9^

Nos dois grupos estudados, DI e DT, as complicações ocorreram majoritariamente na primeira semana após a cirurgia, sendo compatível com dados da literatura consultada.^10 , 11^ No nosso estudo, a taxa global de complicações foi de 27,5% e a taxa de complicação respiratória de 5%. Na literatura, a perda aérea prolongada ocorre, em média, em apena 1,9% dos pacientes, sendo menor do que o observado no nosso estudo, de 5%. Apresentamos taxa de 5% de infecção de parede torácica em cada grupo, semelhante à literatura, em que Akçali et al.,^4^ relataram taxa de 3,3%, também em pacientes submetidos à toracotomia poupadora lateral. Nossos dois grupos apresentaram taxas iguais, o que demonstra que a técnica proposta não aumenta o risco de infecção da ferida operatória.

Ocorreram dois óbitos, sem que houvesse significância estatística. Esses dois pacientes apresentavam comorbidades graves, e os estado clínico dos dois se agravou no período pós-operatório. Acreditamos que os óbitos decorreram de complicações não relacionadas à drenagem.

O fato de as complicações não apresentarem significância estatística entre os grupos confirma o que já era esperado, dado que a técnica uniportal já utilizava esse tipo de drenagem sem apresentar prejuízo em relação à VATS multiportal.^12 , 13^ Esses resultados demonstram a segurança dessa técnica e também que o dreno se mantém pérvio quando inserido de forma proposta. A drenagem pelo mesmo espaço intercostal propicia a preservação do outro espaço, que seria incisado. Além disso, ela evita o risco de lesar um segundo feixe neurovascular e também proporciona uma incisão a menos na camada muscular.

Em nosso estudo, o período de acompanhamento dos pacientes foi curto, de apenas 30 dias. Por isso, seria necessário um tempo mais longo de acompanhamento para confirmar se a DI se manteria semelhante a DT no que tange à morbidade pós-operatória. Esse seguimento mais longo também seria fundamental para comparação nas taxas de dor crônica em ambos os grupos. Além disso, o número de pacientes estudados (apenas 40), não permite definir alguma superioridade da técnica em relação à DT.

## CONCLUSÃO

A drenagem torácica pela toracotomia não foi inferior à drenagem tradicional em pacientes submetidos à toracotomia poupadora lateral. Não foi observado aumento no tempo de internação, no tempo de drenagem, no débito dos drenos, na escala de dor, no uso de analgésicos e nas complicações em relação à drenagem tradicional.
